# Humoral autoimmune response to nucleophosmin in the immunodiagnosis of hepatocellular carcinoma

**DOI:** 10.3892/or.2015.3854

**Published:** 2015-03-16

**Authors:** MEI LIU, ARMANDO VARELA-RAMIREZ, JITIAN LI, LIPING DAI, RENATO J AGUILERA, JIAN-YING ZHANG

**Affiliations:** 1 Department of Biological Sciences and Border Biomedical Research Center, The University of Texas at El Paso, El Paso, TX 79968, USA; 2 Beijing You’an Hospital, Capital Medical University, Beijing 100069, P.R. China

**Keywords:** hepatocellular carcinoma, nucleophosmin, tumor-associated antigen, autoantibody, immunodiagnosis

## Abstract

Hepatocellular carcinoma (HCC) is a worldwide prevalent cancer with an exremely poor prognosis. Detection of serum α-fetoprotein (AFP) and liver imaging techniques are the conventional methods used clinically for the identification of this malignancy. However, these techniques are not reliable for early diagnosis, and particularly the sensitivity and specificity of AFP in HCC diagnosis are not optimal. Therefore, there is an urgent need for the development of more sensitive and specific methods that can improve AFP quantification in the early detection of HCC. In the present study, autoantibody responses to nucleophosmin (NPM1) in HCC patients were evaluated by enzyme-linked immunosorbent assay (ELISA), western blotting and indirect immunofluorescence. Immunohistochemistry (IHC) with tissue array slides was also performed to analyze protein expression of NPM1 in HCC and control tissues. The prevalence of autoantibodies against NPM1 was 22.4% (17/76) in HCC, which was significantly higher than that in sera from patients with liver cirrhosis (LC), chronic hepatitis (CH) and systemic lupus erythematosus (SLE) (P<0.01). The average titer of autoantibodies against NPM1 in HCC sera was higher compared to that in LC, CH, SLE and normal human sera (NHS) (P<0.01). In addition, anti-NMP1 autoantibodies were detected in sera from several HCC patients with serial bleeding samples. A stronger reactive band corresponding to NMP1 was visualized in the western blot analyses, utilizing sera from patients 3–6 months before the clinical diagnosis of HCC. Our data indicate that NPM1 and the anti-NPM1 system may have potential as an early-stage biomarker for HCC screening and diagnosis.

## Introduction

Hepatocellular carcinoma (HCC) is one of the most common types of cancer and the third cause of cancer-related mortality worldwide. The survival expectancy is usually no more than 6 months for HCC patients due to the lack of sensitive detection method at the early stage of cancer. Although serum α-fetoprotein (AFP) is the most commonly used biomarker to detect HCC in clinical screening, its sensitivity and specificity are limited. There are still ~40% of HCC patients that cannot be identified using this approach. HCC patients with small tumors or with well-to-moderately differentiated tumors may not have a high level of serum AFP. Therefore, it is necessary to identify other sensitive biomarkers to ensure a more accurate HCC diagnosis. In the past decade, many studies have demonstrated that tumor-associated antigens (TAAs) and anti-TAA antibodies may be useful biomarkers for the diagnosis of certain types of cancer. The identification of TAAs and anti-TAA antibodies may become a useful tool for HCC diagnosis, focusing on early facets of the disease.

Nucleophosmin (NPM1), also known as nucleolar phosphoprotein B23 or numatrin, is a member of the nucleoplasmin family, which is a ubiquitously expressed nucleolar protein that localizes mainly to the nucleoli, yet also shuttles in and out of the nucleolus, and also between the nucleus and the cytoplasm (1,2). It is associated with nucleolar ribonucleoprotein structures, forming complexes with single-stranded nucleic acids. NPM1 plays multiple roles, including genomic stability, tumorigenesis (3,4), ribosome biogenesis (5,6), centriole replication (3,7,8), cell aging, signal transduction, intracellular protein transport, protein localization and oligomerization, endodeoxyribonuclease activity, activation of the NF-κB transcription factor, DNA repair (9), cell proliferation (10), ribosome assembly (11), response to nucleolar stress (12) and apoptosis (13). Additionally, NPM1 functions as regulator of the tumor-suppressor proteins p53 (14–18) and p14ARF (19), and is also required for the maintenance of genomic stability. NPM1 protein expression increases rapidly in the early G1 phase during mitosis (20). Mutations of the NPM1 gene resulting in the expression of a cytoplasmic mutant protein, NPMc^+^, are the most frequent genetic abnormalities found in acute myeloid leukemia (21). NPM1 is frequently overexpressed, mutated, rearranged and deleted in human cancer cells, therefore it is regarded as a tumor marker (22).

A previous study demonstrated that NPM1 is expressed weakly in normal hepatocytes and is highly expressed in liver cancer cells with a clear correlation between enhanced NPM1 expression and increased tumor grade and consequently poor prognosis (23). Therefore, there is the possibility for NPM1 to be a TAA biomarker for early HCC diagnosis. In the present study, we used an ELISA immunoassay, western blotting, an indirect immunofluorescence assay and immunohistochemistry with a tissue array to evaluate and validate whether the anti-NPM1 autoantibody in patient sera can be used as a novel biomarker for the detection of HCC.

## Materials and methods

### Sera and general information

The sera comprised in the present study were obtained from the serum bank of the Cancer Autoimmunity and Epidemiology Research Laboratory at the University of Texas at El Paso (UTEP), and their sources were as follows: 76 from patients with HCC, 30 from patients with liver cirrhosis (LC), 30 from patients with chronic hepatitis (CH), and 43 from patients with systemic lupus erythematosus (SLE) as well as 89 normal human sera (NHS). In addition, 18 sera from three HCC patients with serial bleeding samples were also tested in this study. The present study was approved by the Institutional Review Board of UTEP and collaborating institutions.

All HCC patients were diagnosed according to the criteria described in a previous study (24), and had not received treatment with any chemotherapy or radiotherapy. Patients with CH and LC were followed up at least 18 months after collecting blood to exclude individuals with primary biliary cirrhosis and asymptomatic or clinically undetectable HCC. Normal human sera were collected from individuals at the same locality during annual health examinations, who had no obvious evidence of malignancy. Of the 76 HCC patients, 50 (65.8%) were male, and 26 (34.2%) were female. Mean age was 57.0±11.2 years (range, 23–77 years). Fifty-two (68.4%) patients were positive for hepatitis B virus (HBV), 6 (7.9%) patients for hepatitis C virus (HCV) and 4 (5.3%) for both HBV and HCV. Forty-eight (63.2%) had a previous history of CH, 13 (17.1%) patients had a previous history of LC and 9 (11.8%) patients had no previous history for either CH or LC. Seventy-one (93.4%) patients were histologically confirmed. Based on the general diagnostic rules for liver cancer, 23 (30.3%) patients were in clinical stage I, 14 (18.4%) in stage II, 24 (31.6%) in stage III, 8 (10.3%) in stage IV, respectively, and 5 (6.7%) patients had no available data concerning clinical stage. In the present study, 62 sera were available for AFP testing. The AFP test kit was provided by GenWay Biotech (San Diego, CA, USA). The results showed that 61.3% (38/62) of the sera had abnormal AFP levels (>100 ng/ml) whereas 24 (38.7%) had normal levels (<100 ng/ml).

### Recombinant proteins and antibodies used in the present study

NPM1 construct GFP-NPM1 WT (plasmid ID: 17578) was purchased from Addgene Inc. (Cambridge, MA, USA), and then subcloned into the pET28a vector to express the fusion protein with an N-terminal 6X histidines and T7 epitope tags. The recombinant protein expressed in *Escherichia coli* BL21 (DE3) was purified using nickel column chromatography. Polyclonal anti-NPM1 rabbit antibody and monoclonal anti-β-actin mouse antibody were obtained from commercial sources (Cell Signaling Technology, Inc., Danvers, MA, USA). Horseradish peroxidase (HRP)-conjugated goat anti-human IgG, HRP-conjugated goat anti-rabbit IgG, HRP-conjugated goat anti-mouse IgG and FITC-conjugated goat anti-human IgG were purchased from Santa Cruz Biotechnology, Inc. (Santa Cruz, CA, USA). Anti-rabbit IgG Fab2 (Alexa Fluor 488) was purchased from Cell Signaling Technology, Inc.

### Enzyme-linked immunosorbent assay (ELISA)

Standard protocol for ELISA was used as described in our previous studies (25,26). In brief, a 96-well microtiter plate (LLC; Immunochemistry Technologies, Bloomington, MN, USA) was coated overnight at 4°C with recombinant NPM1 protein at a final concentration of 0.5 *μ*g/ml in phosphate-buffered saline (PBS). The antigen-coated wells were blocked with gelatin post-coating solution at room temperature for 2 h. Human sera diluted at 1:100 with were added to the antigen-coated wells and incubated for 2 h at room temperature, washed and then incubated with HRP-conjugated goat anti-human IgG (Caltag Laboratories, San Francisco, CA, USA) at a 1:4,000 dilution. The substrate 2,2-azino-bis-3-ethylbenzothiazoline-6-sulfonic acid (ABTS; Sigma-Aldrich, St. Louis, MO, USA) was used to detect the immune complexes. The average optical density (OD) value at a wavelength of 405 nm was used for data analysis. The cut-off value designating a positive reaction was the mean optical density of 90 normal human sera plus 3 standard deviations (SD).

### Western blotting

Denatured recombinant NPM1 protein was electrophoresed on 10% SDS-PAGE and transferred to a nitrocellulose membrane. After blocking in PBS with 5% non-fat milk and 0.05% Tween-20 for 1 h at room temperature, the nitrocellulose membranes were incubated overnight at 4°C with a 1:200 dilution of human sera, a 1:500 dilution of polyclonal anti-NPM1 antibody or a 1:500 dilution of monoclonal anti-β-actin mouse antibody, separately. HRP-conjugated goat anti-human IgG, HRP-conjugated goat anti-rabbit IgG and HRP-conjugated goat anti-mouse IgG were subsequently applied as secondary antibody at a 1:10,000 dilution. The ECL kit was used to detect immunoreactive bands according to the manufacturer's instructions (Thermo Scientific, Waltham, MA, USA).

### Indirect immunofluorescence assay (IIFA) and confocal microscopy

An indirect immunofluorescence assay was performed on Hep2 antinuclear antigen tissue slides (Bion Enterprises, Des Plaines, IL, USA). The human sera were diluted at 1:80 in PBS, pH 7.4 and incubated with the slides for 30 min at room temperature. After extensive washing, the slides were incubated with fluorescein isothiocyanate (FITC)-conjugated goat anti-human IgG secondary antibody (Santa Cruz Biotechnology, Inc.) or anti-rabbit IgG Fab2 (Alexa Fluor 488) as secondary antibody diluted 1:100 in PBS for 1 h at room temperature. The slides were washed two times with PBS before adding a drop of mounting media containing 1.5 *μ*g/ml 4',6'-diamidino-2-phenylindole (DAPI) (Vector Laboratories, Inc., Burlingame, CA, USA). To prevent photobleaching, the latest steps were performed in the dark. The slides were then examined under fluorescence microscopy (LSM 700 confocal microscope; Zeiss) at x400 magnification, and Zen 2009 software was used for image capture and analysis.

### Absorption of the antibodies with recombinant protein

The diluted human sera (1:80) were incubated overnight at 4°C with recombinant NPM1 protein, at a final concentration of 0.03 *μ*g/*μ*l, and then centrifuged at 10,000 × g for 10 min. The antigen absorbed supernatant was used for the immunofluorescence assay.

### Immunohistochemistry (IHC) with tissue array slides

Liver cancer tissue array slides with normal tissue controls (38 cases/80 cores, including pathological diagnosis and pathological grades, 10 normal liver tissues as controls) were purchased (US Biomax, Inc., Rockville, MD, USA), and used to detect the expression of the NPM1 protein. Tissue array slides were deparaffinized with xylene and dehydrated with ethanol. Antigen retrieval was performed by microwave-heating methods in Trilogy™ pretreatment solution for 20 min. Avidin/biotin blocking solution was used to prevent nonspecific binding of the antibodies. The tissue sections were incubated with polyclonal anti-NPM antibody (1:50 dilution) overnight at 4°C. HRP detection system (HRP streptavidin label and polyvalent biotinylated link) and diaminobenzidine (DAB) substrate kit were used as detecting reagents. After counterstaining with hematoxylin, the sections were dehydrated and mounted. The slides were observed by light microscopy (Leica DM1000, Germany).

### Statistical analysis

The mean OD value of each group of patient sera was compared using the Mann-Whitney U test; the frequency of autoantibody to TAAs in each group of patient sera and the expression profile of NPM1 in the liver cancer and normal tissue groups were compared using the Chi-square (χ^2^) test with Fisher's exact test, and two levels of significance (0.05 and 0.01) were used.

## Results

### Frequency and titer of autoantibodies against NPM1 in HCC

**Figure 1:**
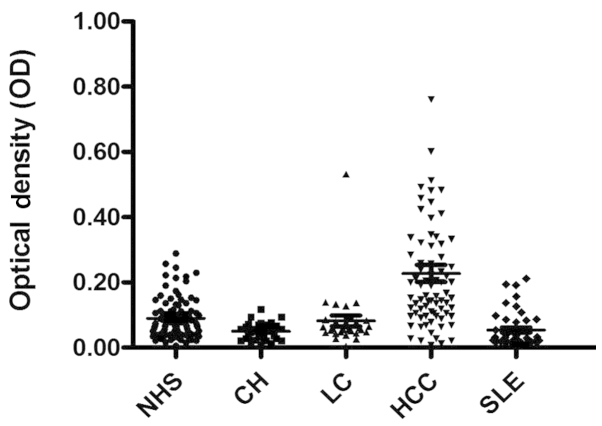
Titer of the autoantibody against NPM1 in human sera by ELISA. The range of antibody titers to NPM1 was expressed as optical density (OD) obtained from ELISA. The mean ± 3SD of NHS are shown in relationship to all serum samples. The titer of anti-NPM1 in HCC was much higher than that in other types of sera (P<0.01). NPM1, nucleophosmin; NHS, normal human sera; CH, chronic hepatitis; LC, liver cirrhosis; HCC, hepatocellular carcinoma; SLE, systemic lupus erythematosus.

**Figure 2:**
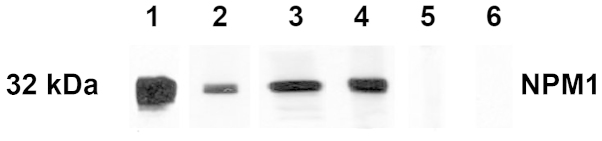
Western blot analysis with representative sera in ELISA. Lane 1, the polyclonal anti-NPM1 antibody was used as positive control. Lanes 2–4, three representative HCC sera which were positive in ELISA also had strong reactivity with NPM1 recombinant protein in western blot analysis. Lanes 5 and 6, two randomly selected NHS had negative reactivity to NPM1 recombinant protein. NPM1, nucleophosmin; HCC, hepatocellular carcinoma; NHS, normal human sera.

**Figure 3:**
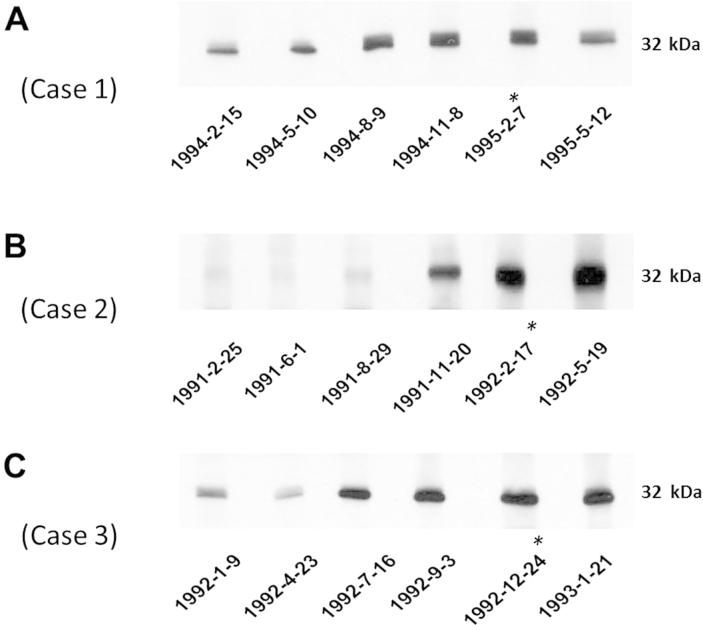
Autoantibody to NPM1 in serial serum samples from three HCC patients (cases 1–3). (A) The first HCC patient (case no. 1) was diagnosed on 02/07/1995 with strong expression of the anti-NPM1 autoantibody. A total of six serum samples within more than one year of time span was collected from this HCC patient and the NPM1 reactive bands were present in all of them. It became stronger at 6 months before HCC was detected. (B) A total of six serum samples were acquired from the second HCC patient (case no. 2). The anti-NPM1 autoantibody appeared at 3 months before the date (02/17/1992) of HCC diagnosis. There were almost no NPM1 reactive bands before 11/20/1991. (C) The third HCC patient (case no. 3) was diagnosed at the date of 12/24/1992, with a strong NPM1 reactive band. During a one-year period (01/09/1992–01/21/1993), six serum samples collected from this patient showed a gradually increased reactive band for NPM1. Clearly, a stronger reactive band was observed in the serum at 6 months before the diagnosis of HCC. NPM1, nucleophosmin; HCC, hepatocellular carcinoma.

The full-length recombinant NPM1 protein was used as a coating antigen in ELISA to screen autoantibodies against NPM1 in sera from patients with HCC, LC, CH, SLE and as well as NHS. In total, 76 sera from patients with HCC, 30 from LC, 30 from CH and 89 sera from normal human individuals were used in the present study. As shown in Table I, the prevalence of the autoantibody against NPM1 was 22.4% (17/76) in HCC, which was significantly higher than that in LC, CH, SLE and NHS (P<0.01). The titer of the anti-NPM1 antibodies in human sera is shown in Fig. 1. The average titer of the autoantibody against NPM1 in HCC sera was higher than that in LC, CH, SLE and NHS (P<0.01). The ELISA results were also confirmed by western blot analysis. Fig. 2 shows that representative HCC sera with a positive reaction to NPM1 in ELISA also had strong reactivity in the western blotting compared to the normal sera. The autoantibody to NPM1 in serial serum samples from three HCC patients (case nos. 1–3) was also tested. The western blotting results are shown in Fig. 3. In HCC case 1 and HCC case 3, the anti-NPM1 autoantibody was stronger at 6 months before HCC was detected. In HCC case 2, the anti-NPM1 autoantibody appeared at 3 months before the date of the HCC diagnosis.

Of the 76 HCC sera, 62 were tested for the presence of both the anti-NPM1 autoantibody and AFP; 38 sera (61.3%) had an AFP level >100 ng/ml, and 15 sera (24.2%) were positive for the anti-NPM1 autoantibody. When both the anti-NPM1 autoantibody and AFP (>100 ng/ml) were simultaneously used as diagnostic markers, 43 (69.4%, AFP >100 ng/ml) of the 62 HCC sera were positive. Out of the 17 sera with a normal level of AFP, 5 (29.4%) were positive for the anti-NPM1 autoantibody.

### Detection of an intense nuclear staining pattern in Hep2 cells by indirect immunofluorescence assay with representative positive HCC sera

**Figure 4:**
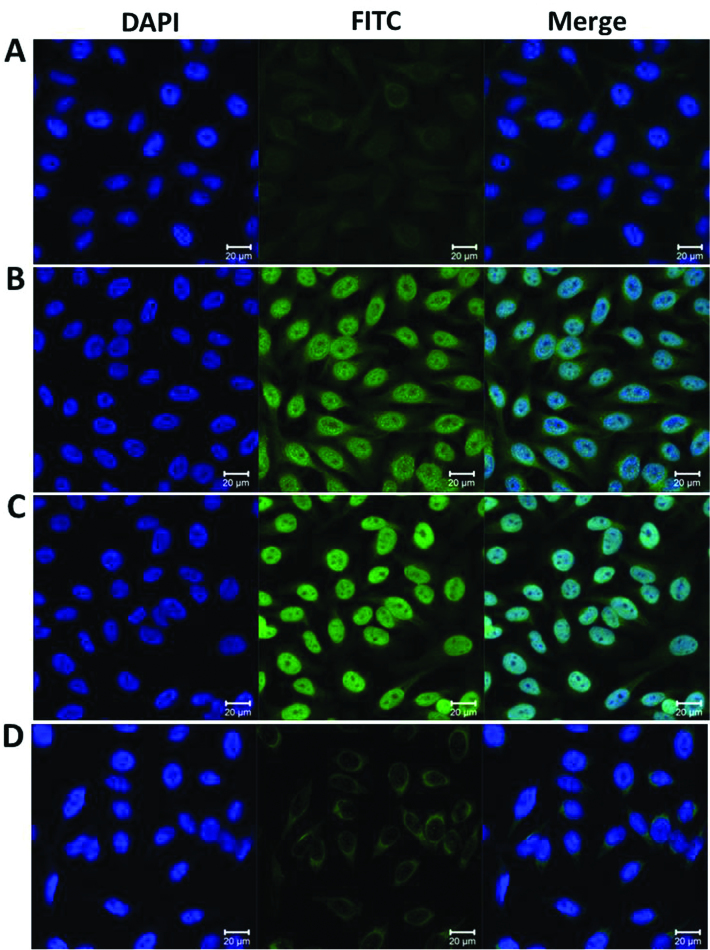
Representative immunofluorescence staining pattern of anti-NPM1 autoantibody-positive HCC serum. (A) NHS were used as a negative control. (B) Polyclonal anti-NPM1 antibody which showed a nuclear immunofluorescence staining pattern was used as a positive control (green signal). (C) Representative anti-NPM1 autoantibody-positive HCC serum demonstrated an intense nuclear staining pattern. (D) The same HCC serum used in panel C was pre-absorbed with recombinant NPM1. The nuclear fluorescent staining was significantly reduced. Scale bars, 20 *μ*m. NPM1, nucleophosmin; HCC, hepatocellular carcinoma; NHS, normal human sera. DNA was stained with DAPI (blue) (left panels).

To further confirm the reactivity of the auto antibody in HCC sera to NPM1 and the intracellular location of NPM1, commercially purchased Hep2 cell slides were used in an indirect immunofluorescence assay to detect HCC sera with anti-NPM1 positivity in ELISA. As shown in Fig. 4, a representative anti-NPM1-positive HCC serum had an intense nuclear staining pattern, which was similar in fluorescent staining pattern and cellular location to that shown by the polyclonal anti-NPM1 antibody. This fluorescence signal was significantly reduced when the same HCC serum was pre-absorbed with recombinant NPM1 protein.

### Expression of NPM1 in liver cancer and normal hepatic tissues by immunohistochemistry

**Figure 5:**
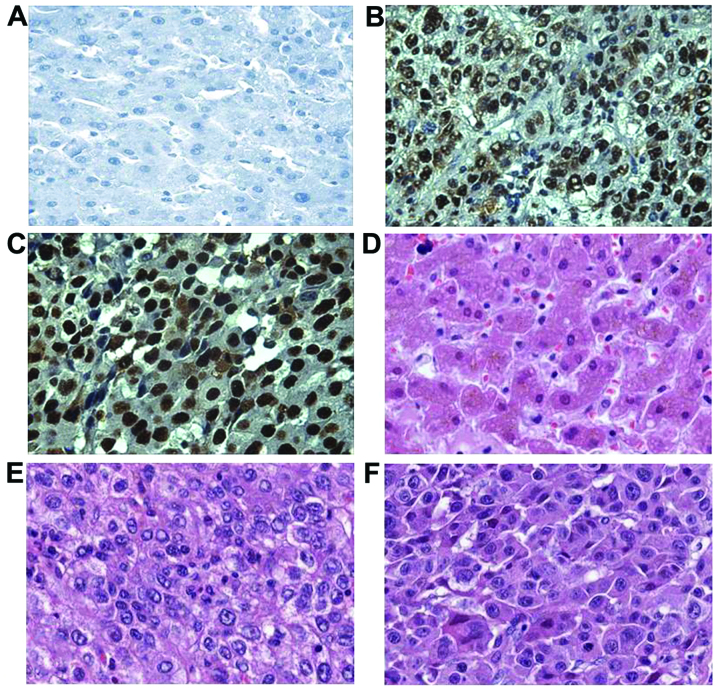
Expression of NPM1 in liver cancer and normal hepatic tissues by immunohistochemistry. The polyclonal anti-NPM1 antibody was used as a primary antibody to detect the expression of NPM1 in liver cancer and normal hepatic tissues. (A) Normal hepatic tissue with negative staining. (B) HCC tissue (grade II) with a positive staining signal. (C) HCC tissue (grade III) with a strong positive staining signal. (D) A normal hepatic tissue with H&E staining signal. (E) HCC tissue (grade II) with H&E staining signal. (F) HCC tissue (grade III) with H&E staining signal. Grade II, moderately differentiated, cells appear slightly different than normal. Grade III, poorly differentiated, cells appear abnormal and tend to grow and spread more aggressively. NPM1, nucleophosmin; HCC, hepatocellular carcinoma; H&E, hematoxylin and eosin.

In the present study, the expression profile of NPM1 in liver cancer and normal liver tissues was examined by immunohistochemistry with tissue array slides. Tissue array slides were commercially available for the present study; including 30 liver tissues from HCC patients and 10 from normal hepatic tissue donors. The polyclonal anti-NPM1 antibody was used as the primary antibody to detect the expression of NPM1 in both liver cancer and normal tissues. As a result, 22 of the 30 HCC tissues were positively stained (73.3%). Among these 22 positively stained tissues, 5 were strongly stained, whereas 17 were moderately stained. In contrast, one of the 10 normal hepatic tissues exhibited a positive staining pattern (10%). The characteristics of patients and NPM1 expression in liver cancer are shown in Table II. The expression of NPM1 in liver cancer and that in normal tissues are shown in Fig. 5.

## Discussion

Antigenic changes in cancer cells can be recognized by the immune system of patients themselves as autoantibody responses to proteins involved in malignant transformation. These autoantibodies, which have been called ‘reporters’ from the immune system, can be used as probes in immunoscreening cDNA expression libraries and immunoproteomics to isolate, identify and characterize potential TAAs (27,28). Many studies have demonstrated that serological screening of autoantibodies to TAAs is useful in the diagnosis of certain types of cancer in an early stage of their development. In recent years, a large number of TAAs in cancer have been identified using these approaches. Some of them have the possibility to be used as biomarkers in cancer immunodiagnosis (29–31). It is well demonstrated that cancer has long been recognized as a multistep process, which involves not only genetic changes conferring growth advantage but also factors that disrupt the regulation of growth and differentiation (32,33). It is possible that some of these factors could be identified and their functions evaluated with the aid of autoantibodies arising during tumorigenesis. In our previous study, certain proteins such as p62, c-Myc, p53, cyclin B1, survivin, p16, RalA, Koc, IMP-1, Sui1, HCC1, GRP78 and p90, were evaluated and validated as TAAs in HCC, and autoantibodies against these TAAs have been detected in sera from patients with HCC (34,35). In another study, it was suggested that autoantibodies against these TAAs appear to be supplementary serological markers for the diagnosis of HCC in AFP-negative cases (36). Our previous studies revealed that the sensitivity and specificity of autoantibodies to a single TAA as a diagnostic marker in HCC are currently not high enough for the diagnosis of HCC (34). Therefore, it is necessary to continue the study to identify more TAAs for HCC. Another technique to solve this problem is to combine all the known HCC-related TAAs to develop a mini-array of multiple TAAs to detect autoantibodies simultaneously in order to enhance the sensitivity and specificity (37). One of our previous studies showed that the final cumulative prevalence of autoantibodies to 10 TAAs can reach a sensitivity of 66.2% in sera from patients with HCC (34).

NPM1 is a multifunctional protein involved in a complex network of interactions, yet its actual role in oncogenesis is controversial (22). The NPM1 gene is mutated or rearranged in a number of hematological disorders, and it was considered as the most frequently mutated gene in acute myeloid leukemia (38). Yet, its role in leukemogenesis remains unclear. A knock-in NPM1 mutation in mice was found to result in myeloproliferation and denoted a disruption in the hematopoietic microenvironment (39). Knockdown of NPM1 by RNA interference inhibited cell proliferation and induced apoptosis in a leukemic cell line (40). Yet, such changes have not been detected in solid cancers. However, overexpression of NPM1 is often found in many types of solid tumors (41–43). Another study uncovered the critical role of NPM1 in the regulation of colon cancer cell migration and invasion, and NPM1 may serve as a potential marker for the prognosis of colon cancer patients (44). Some results support a tumor-suppressive role for NPM1 in breast cancer (45). Experiments with NPM1-knockout cells indicate a tumor-suppressor function for NPM1, both through its role in the maintenance of genomic stability and in the regulation of the alternative reading frame (ARF) tumor-suppressing pathway. Although genetic analysis implicates NPM1 in tumorigenesis, it is still not clear whether NPM1 may operate either as an oncogene or a tumor-suppressor gene, perhaps with dual functions (22). Our present study indicated that more than 20% of HCC sera showed an immune response to NPM1 recombinant protein. The mean titer of autoantibodies against NPM1 in sera from patients with HCC was significantly higher than that in LC, CH, SLE and normal individuals. It is known that LC and CH are common precursor conditions of HCC. Clinical surveillance for high-risk individuals, such as CH and LC patients, is important for detecting early-stage HCC. During transition to malignancy, some HCC patients develop autoantibodies that are not present during the preceding chronic liver disease phase. These types of autoantibodies may have value for the diagnosis of HCC patients at an early stage of tumorigenesis. Anti-NPM1 autoantibodies are potential biomarkers for the immunodiagnosis of HCC due to the low positive rate in LC, CH, SLE and normal individuals and the higher positive rate in HCC patients. As a control group, sera from patients with SLE which is an autoimmune disease were also detected for the presence of anti-NPM1 antibodies. The data suggested that anti-NPM1 autoantibodies are not related to autoimmune diseases such as SLE and other chronic liver diseases, and these antibodies may have close relevance to HCC.

The results in the present study also indicated that when both the autoantibody against NPM1 and AFP are used as diagnostic markers simultaneously, sensitivity can reach 69.4%, which is much higher than that when using either anti-NPM1 or AFP as a marker. It is more important to note that, in HCC patients with AFP negativity, there were still 29.4% of patients who could be positively detected with the anti-NPM1 autoantibody. Our data indicate that the anti-NPM1 autoantibody may be a good supplemental marker of AFP in HCC diagnosis. In order to test dynamic changes in anti-NPM1 autoantibodies during malignant transformation from chronic liver diseases to liver cancer, the sera from three HCC cases with serial bleeding serum samples were available in our laboratory, and were tested in the present study. Notably, six serum samples collected from each patient showed a gradually increased reactivity to NPM1. Western blot analysis showed that stronger reactive bands were observed in the serum at 3–6 months before the clinical diagnosis of HCC. These results indicated that the titer of the autoantibodies against NPM1 in HCC sera increased at 3–6 months before the diagnosis of HCC, and the autoantibodies against NPM1 in HCC sera may be potential biomarkers for early-stage HCC screening and diagnosis.

Taken together, much evidence suggests that NPM1 is highly expressed in various tumors and is correlated with the stage of tumor progression and poor prognosis (23). Overexpression of NPM1 mRNA is independently associated with the recurrence of bladder carcinoma and progression to a more advanced stage of disease. A higher NPM1 level was linked to more advanced tumor stages, grades, poor prognosis and likelihood of recurrence (41,46). In a study of human breast cancer, NPM1 was identified as an estrogen-regulated protein associated with acquired estrogen independence by two-dimensional gel electrophoresis analyses (47). Yun *et al* found that NPM expression was significantly higher in HCC than in non-malignant hepatocytes, while it was weakly expressed in hepatocytes from a 5-month-old embryo and in stationary hepatocytes of healthy adults (48). In the present study, we also found that NPM1 expression was markedly increased in liver cancer tissues. Grade III HCC tissues exhibited a stronger positive signal than HCC tissues with grade II cancer, when using immunohistochemistry approach with HCC tissue array slides.

Biological effects such as increased cell growth and proliferation, and inhibition of differentiation and apoptosis are characteristics of neoplastic transformation. All of these biological effects have been demonstrated to be correlated with NPM1 overexpression in tumor cells. Due to the diversity of cellular activities exhibited, NPM1 is a key player with dual functions of either a potential oncogene or a potential tumor suppressor. Deregulation of NPM1 expression and/or localization could therefore contribute to tumorigenesis through different mechanisms (22). The functions of overexpression of NPM1 in HCC are not clear, yet our data support the conclusion that NPM1 has a tight relationship with the occurrence of HCC. Many studies have demonstrated that NPM1 is a ubiquitously expressed nucleolar protein that localizes mainly to the nucleoli, but also shuttles in and out of the nucleolus, and also between the nucleus and the cytoplasm. This complex mechanism of NPM1 may be the reason why NPM1 is involved in multiple cellular functions. The shuttling activity of NPM1 and its proper subcellular localization may be crucial for cellular homeostasis. In the present study, NPM1 was found in the nucleus of cells. An IHC study with an HCC tissue array further verified that the cellular localization of NPM1 is also in the nucleus. In contrast, in a study on acute myelogenous leukemia, a mutant NPM1 counterpart (NPMc^+^) was aberrantly localized in the cytoplasm of leukemic blasts (49). The different locations of NPM1 in cancer cells may indicate a different role of NPM1 in the tumorigenesis pathway. Although much evidence demonstrates that NPM1 expression correlates with clinical parameters of cancer patients, the detailed role of NPM1 in cancer progression is largely unknown.

In summary, the data from the present study provide further evidence that NMP1 can be used as a potential TAA, and the anti-NMP1 autoantibody is a useful immunodiagnostic biomarker for the early detection of HCC. The underlying mechanism of how NPM1 induces humoral immune response in HCC patients, and how it is involved in the tumorigenesis of HCC still require investigation.

## Figures and Tables

**Table I:** Frequency of the autoantibody against NPM1 in human sera by ELISA.

Type of sera	No. tested	Autoantibody to NPM1 (%)
HCC	76	17 (22.4)^a^
LC	30	1 (3.3)
CH	30	0
SLE	43	0
NHS	89	1 (1.1)

Cut-off value, mean ± 3 SD of NHS.

a P<0.01, relative to NHS, SLE, CH and LC. HCC, hepatocellular carcinoma; LC, liver cirrhosis; CH, chronic hepatitis; SLE, systemic lupus erythematosus; NHS, normal human sera; NPM1, nucleophosmin.

**Table II:** Characteristics of the patients and NPM1 expression in liver cancer.

Variable	Frequency	%
Age (years)		
≥60	7	23.3
<60	23	76.7
Gender		
Male	20	66.7
Female	10	33.3
Grade		
2	15	50.0
3	15	50.0
Normal liver tissue		
Negative	9	90.0
Positive	1	10.0
Liver cancer		
Negative	8	26.7
Positive	22	73.3^a^

a P<0.01, liver cancer compared to normal tissue. NPM1, nucleophosmin.
